# 

**Published:** 1888-12-01

**Authors:** 


### CONTENTS.

**Are We a Nation of Liars?** - 129

**The Student's Column.**—London University Pass Lists - 130

**Charing Cross and Other Hospitals.** By the Right Hon. the EARL of DERBY - 131

**The Case for Special Hospitals.** By Mr. LENNOX BROWNE, F.R.C.S.E., and Mr. HENRY POWER, F.R.C.S. - 132

**The Doctor's Pulpit.**—Present-Day Problems - 133

**Glances at the Insane in Other Lands.** I. New South Wales - 134

**Voyages in Vacation.**—X. Impressions of St. Petersburg - 135

**Notes and News** - 135

**Everybody's Page** - 138

**Annotations.**—Hot Bottles in Bed.—The Doctor and His Fee.—Tea.—Wet Feet - 139

**Women and Their Victims** - 140

**Dundee Royal Infirmary.** By R. NEAVES M'COSH, M.D., the Medical Superintendent - 140

**False Impressions.**—By CAROLINE FOTHERGILL. Chap. IX. 141

**Physiology and Its Uses.**—The Blood - 143

**Scraps and Gleanings—Amusements and Relaxation** 144

### EXTRA SUPPLEMENT.—THE NURSING MIRROR.

**En Passant.**—Charing Cross—The Price of Attention—Lady Nurses—British Nurses' Association—News from Abroad—The Women's Jubilee Offering—Trained or Not? - xxxiii

**Original Articles.**—One Way of Nursing. Concluded - xxxiv

**Rules for Nurses** - xxxiv

**Wages and Washing.** Part I. - xxxv

**Everybody's Opinion**—Mildmay Nurses—Nurses and Presents—A Speciality—Correcting an Error—Night Duty, etc. - xxxvi

**The Nurses' Bookshelf** - xxxvi

**Appointments—Vacancies—Notes and Queries** - xxxvi

